# Transcriptome of *Taenia solium* during *in vitro* cyst activation and initial growth into the tapeworm stage

**DOI:** 10.1038/s41597-025-05141-2

**Published:** 2025-05-17

**Authors:** David Castaneda-Carpio, Renzo Gutierrez-Loli, Jose Maravi-Jaime, Segundo W. Del Aguila, Valeria Villar-Davila, Luz M. Moyano, Rafael Tapia-Limonchi, Stella M. Chenet, Cristina Guerra-Giraldez

**Affiliations:** 1 Laboratorio de Proliferación Celular y Regeneración, Facultad de Ciencias e Ingeniería, Universidad Peruana Cayetano Heredia, Lima, Peru; 2 Centro de Salud Global, Tumbes, Universidad Peruana Cayetano Heredia, San Martín de Porres, Peru; 3 Escuela Profesional de Medicina Humana, Universidad Nacional de Tumbes, Tumbes, Peru; 4 Instituto de Investigación de Enfermedades Tropicales, Universidad Nacional Toribio Rodríguez de Mendoza de Amazonas, Chachapoyas, Peru; 5 Facultad de Medicina, Universidad Nacional Toribio Rodríguez de Mendoza de Amazonas, Chachapoyas, Peru; 6 Present Address: Toulouse Institute for Infectious and Inflammatory Diseases, Université de Toulouse, CNRS, Inserm, Toulouse, 31300 France; 7 Present Address: Villanueva Lab, Molecular and Cellular Oncogenesis Program, The Wistar Institute, Philadelphia, PA 19104 USA

**Keywords:** Parasitic infection, Parasite development

## Abstract

The cestode *Taenia solium* develops as a tapeworm solely in the human intestine, starting from a larva (cyst). Upon maturing, it produces hundreds of thousands of infectious eggs. When ingested by pigs or humans, the eggs develop as cysts that lodge in various tissues, including the brain, leading to neurocysticercosis. Despite advances in understanding cestode biology through genomic and transcriptomic studies, particularly in model organisms, much remains unknown about the activation of *T. solium* cysts in the human digestive tract and the events that drive the development into adult worms—the stage responsible for dispersing the parasite. We present a transcriptome generated by Next Generation Sequencing from *T. solium* cysts activated in culture and collected at three different *in vitro* growth phases, defined by their morphology. Differentially expressed genes and biological processes relevant to activation and growth can be explored with the dataset. The information is valuable for identifying genes that regulate the molecular, metabolic, and cellular events leading to parasite maturation or elements driving its transmission.

## Background & Summary

*Taenia solium* is a parasitic flatworm causing neurocysticercosis and taeniasis. Neurocysticercosis, from ingesting *T. solium* eggs, leads to neurological symptoms and can be fatal. This neglected tropical disease is common in areas with poor sanitation and free-roaming pigs, causing up to 70% of epilepsy cases where it is endemic^1^. The World Health Organization (WHO) identifies *T. solium* as a major cause of death from food-borne diseases, resulting in 2.8 million disability-adjusted life-years (DALYs) globally^2^. Infected animals also cause economic losses for farmers^3^.

*T. solium*’s life cycle involves pigs as intermediate hosts and humans as the only definitive hosts. The adult tapeworm sheds embryonated eggs in human feces. When ingested by pigs or humans, embryos travel mainly to muscles, the heart, and the brain while developing into vesicular cysts^4^. These cysts, if consumed in undercooked pork, are activated by digestive enzymes and bile salts. The activation triggers the evagination of the parasite’s scolex, a process where the scolex, containing hooks and suckers, emerges from the cyst^5^. The evagination is driven by cell proliferation at the neck, where totipotent cells play a crucial role. These cells facilitate the eversion of the scolex, which can then attach to the human intestine^6^. The parasite grows continuously by generating segments (proglottids) that produce eggs, perpetuating the cycle^4,7,8^.

Humans thus carry the parasite’s dispersal form, the tapeworm with mature proglottids. A few carriers can infect many pigs in the rural context of a developing country^9,10^. One Health strategies aim to control the disease by treating pigs and humans^11^. Mathematical models like CystiAgent highlight the importance of reducing the tapeworm’s infectious duration to curb human-to-porcine transmission^9,12^. However, the biology of this stage is the least known, and studying it is challenging, due to ethical and practical issues associated with humans as hosts.

The parasite’s genome^13^ and partial transcriptomes have provided insights into its development^14,15^. Comparative studies have identified common genes and pathways, including whole-transcriptome analyses with Next Generation Sequencing of cestodes *Echinococcus*^16^, *Hymenolepis*^17^, and other *Taenia*^18,19^. Still, the life cycles of these species differ significantly from *T. solium*, leaving a critical gap in understanding *T. solium*’s specific molecular and cellular mechanisms during cyst activation and subsequent development into the adult worm. Single-gene approaches have focused on the early development of bile-activated cysts^20^. Bile salts have long been used to catalyze the *in vitro* transition to the adult stage^21,22^, but the transcriptomic program triggered by this treatment—mimicking cyst activation in the human intestine—has not been characterized.

**Fig. 1:**
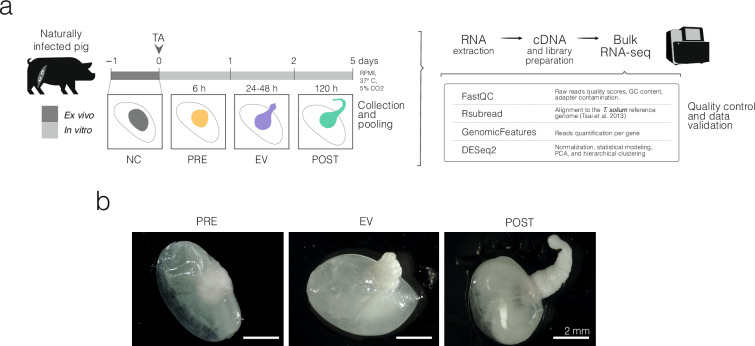
Experimental design and morphological phases of *T. solium* cysts around the evagination of the scolex *in vitro*. (**a**) Overview of the *in vitro* culture and sampling strategy. *T. solium* cysts from naturally infected pigs were cultured in RPMI medium at 37 °C with 5% CO_2_. Taurocholic acid (TA) was added at time zero. Samples collected at three distinct moments correspond to different morphological phases around scolex evagination: PRE (6 h *in vitro*), EV (24–48 h *in vitro*), and POST (120 h *in vitro*). RNA extraction, cDNA synthesis, and bulk RNA sequencing followed. Bioinformatic analyses included quality control (FastQC), alignment (Rsubread), gene quantification (GenomicFeatures), and statistical analysis (DESeq 2). (**b**) Representative images of cysts from each group.

Our aim is to provide information about *T. solium*’s cyst activation and growth into the adult intestinal form. We incubated fresh cysts extracted from naturally infected pig muscle, adding a bile acid, taurocholic acid (TA), for cyst activation. We empirically determined three morphological phases—one before and two after scolex evagination—, collected samples at each phase, and performed RNA-Seq. We assessed the quality of the sequencing reads and mapped them to the *T. solium* reference genome (Fig. 1a).

The dataset^23^ can serve as a starting point for exploring condition-specific gene expression patterns through differential gene expression and gene enrichment analyses. For example, the groups selected for the transcriptome allow discrimination between events happening on parasites before and after TA-induced evagination of the scolex.

Few *Taenia solium* genomes and transcriptomes are available; therefore, reliable functional annotations for each gene are lacking. In the WormBase ParaSite database, only 6,168 (49.5%) have at least one associated Gene Ontology (GO) term, with a mean of 2.499 GO terms per gene. To provide more elements to analyze and compare, we enhanced the data available in public repositories by functional annotation with blast2GO^24^. This added 3,317 newly annotated genes (26.6% of the total 12,467 *T. solium* coding sequences^13^) and 9,821 extra GO terms. Our blast2GO annotation assigned at least one GO term to 9,485 (76.1%) genes present in WormBase ParaSite, with a mean of 15.426 GO terms per gene, leaving only 2,982 (23.9%) genes without annotation. Additionally, we generated a custom annotation package with AnnotationForge^25^ to facilitate over-representation analysis using clusterProfiler^26^, enabling a more detailed exploration of the biological processes active in the parasite.

This dataset^23^ offers the first transcriptome focused on the *T. solium* cyst activation and development into a tapeworm, processes naturally occurring inside the human host. By associating transcripts to phases surrounding cyst activation, the data provides a foundation for future research on gene function in *T. solium* early-adult development and a model to examine scolex evagination and strobilation at multiple molecular, cellular, histological, and morphological levels. Further investigation could uncover regulatory elements and pathways triggering and modulating the maturation of *T. solium* in the human intestine.

## Methods

### Ethics statement

The study protocol was revised and approved by the Animal Care and Use Committee (CIEA, in Spanish) at Universidad Peruana Cayetano Heredia (CIEA certificates 040-09-22, R 040-09-23, and R 040-09-24, valid until August 2025). The CIEA states details for the proper housing, feeding, and handling of animals used in research, including euthanasia. This committee is registered with the Office of Laboratory Animal Welfare of the Department of Health and Human Services of the National Institutes of Health (NIH—USA) with Assurance Number F16-00076 (A5146-01), valid until August 31, 2026.

### Collection of *T. solium* cysts from pig muscle

*Taenia solium* cysts (n = 500) were collected under aseptic conditions at Universidad Peruana Cayetano Heredia’s Global Health Center in Tumbes, Peru. The parasites were excised from the muscle tissue of a naturally infected pig carcass using sterile scalpels, ensuring that the surrounding capsule of inflammatory infiltrate and collagen was removed entirely. Upon collection, cysts were washed three times in sterile 50-mL Falcon tubes with cold transport solution (1X PBS, pH 7.2, with Gibco^TM^ Antibiotic-Antimycotic to the following final concentrations: 100 units/mL penicillin, 100 μg/mL streptomycin, and 250 ng/mL amphotericin B). Each wash involved gently inverting the tube several times over a period of 3 minutes; the solution was replaced between washes. Cysts were transferred to sterile 50-mL Falcon tubes (up to 100 cysts per tube) with at least twice their volume of fresh transport solution and packed in an insulating container that was kept below 12–15 degrees until its arrival by air freight to the university laboratories in Lima.

### *In vitro* scolex evagination and morphology of activated parasites

Cysts arrived at the laboratory approximately 16 hours after collection and were washed three times with transport solution like before, maintaining an aseptic environment. After shipping and washing, most cysts maintained their characteristics: intact, white, or translucent oblong-shaped vesicles without atypical coloration, filled with a clear fluid (Fig. 1b). Cysts were then distributed for different purposes; 84 were used for the present study.

Twelve cysts were placed each in 1 mL of RNAlater® solution (R-0901, Sigma Aldrich) and stored at −20 °C until RNA extraction. These *ex vivo* cysts were defined as non-cultured (NC).

For culture, washed cysts were transferred to culture plates with sterile Pasteur pipettes, working in a laminar flow hood. Each one of 72 cysts was incubated in 1.5 ml filtered sterilized RPMI 1640 medium (pH 7.4), supplemented with 2 g/mL NaHCO_3_ (Merck®, Burlington, MA), 1.6 µM β-mercaptoethanol, and Gibco™ Antibiotic-Antimycotic in the concentrations described above. One cyst per well was placed in three Costar® 24-well clear Tissue Culture-treated surface plates (Corning®, Corning, NY, USA) at 37 °C and 5% CO_2_ in a sterile incubator (VWR Symphony Incubator) for up to five days. The medium of 36 of the 72 cysts included 0.1% taurocholic acid (TA) (Sigma, St. Louis, MO, USA). Therefore, half of the samples were TA+ and half TA-. The medium was replaced every 24 hours. Cysts were collected in three distinct moments: cysts with non-evaginated scolex, after 6 hours *in vitro*, TA+ and TA- (defined as “pre-evagination”: PRE TA+ and PRE TA-); cysts with recently exposed scolex, approximately after 24–48 hours *in vitro* (defined as “recent evagination”: EV TA+ and EV TA-); and cysts having fully evaginated the scolex, already showing some proglottids, after 120 hours *in vitro* (defined as “post-evagination”: POST TA+ and POST TA-). After culture, all parasites were washed with 1X PBS to remove residual media, and preserved at −20 °C in 1 mL RNAlater® solution until RNA extraction. Evagination curves were made along the 120 hours *in vitro*.

### RNA isolation

RNA was extracted from 12 samples (cysts) of each of the seven groups (NC, PRE TA+, PRE TA-, EV TA+, EV TA-, POST TA+, POST TA-; n = 84 cysts) using the Quick-RNA Miniprep Plus Kit (Zymo Research, R1058). Each preserved cyst was placed on a sterile Petri dish, and its vesicle was carefully removed with a sterile scalpel, leaving only the parenchymatous portion (scolex, neck, and eventual proglottids). Samples were independently washed thrice with molecular biology grade water (Molecular grade; Fisher Scientific), then embedded in 600 µL of DNA/RNA Shield (Zymo Research, R1058), and admixed with a sterile tissue homogenizer tip (Omni International, Kennesaw, GA) on ice, followed immediately by a 30-min treatment with 60 mg of Proteinase K (Zymo Research, D3001-2-60). RNA was isolated following the manufacturer’s protocol, with an extra washing step with 400 µL of RNA Wash Buffer (Zymo Research, R1058) before final RNA isolation. The protocol included DNAse I treatment (Zymo Research, E1009-A (250 U)). As recommended by the manufacturer, a mix of 5 µL of DNase I (1 U/µL) and 75 µL of DNA Digestion Buffer (Zymo Research, R1058) was added directly to the silica column matrix (Zymo-Spin™ IIICG Columns) containing processed samples, for a 15-min incubation at room temperature (20–30 °C). RNA yield was measured by spectrophotometry with NanoDrop® (Thermo Fisher Scientific, Waltham, MA, USA), and approximately 300 ng of RNA were loaded into a 1.5% agarose/1% bleach gel to test its integrity by electrophoresis (35 minutes at 100 V)^27^.

### RNA-Seq library construction and sequencing

We constructed 21 libraries, three for each of the seven groups (NC, PRE TA+, PRE TA-, EV TA+, EV TA-, POST TA+, POST TA-). Each library was made from RNA from four cysts, pooling 125 ng of independently extracted RNA for a final 500 ng/library. Following the manufacturer’s instructions, the Illumina® Stranded mRNA Prep Ligation Kit (Illumina, 20040532) was used to construct libraries indexed with the IDT for Illumina RNA Unique dual (UD) Indexes. Briefly, Oligo(dT) RNA purification magnetic beads (RPBX, Illumina, 20040532) allowed for polyA-RNA selection and excluded ribosomal RNA. Fragmentation of purified mRNA, cDNA synthesis of the first and second strands, and ligation of the Illumina RNA UD Indexes Set A followed. The libraries were amplified by 13 PCR cycles and quantified using the Qubit™ dsDNA High Sensitivity (HS) Assay Kit (Thermo Fisher Scientific, Q32851). The library fragment sizes and their integrities were verified on 2% agarose gel electrophoresis.

Each library was diluted to a starting concentration of 1 nM, and the 21 libraries were combined using 10 μL of each diluted library. The combined libraries were denatured and diluted to a final concentration of 1.4 pM before sequencing. Libraries were sequenced for 75 cycles in paired-end mode with a high output kit on the Illumina NextSeq 500 platform (Illumina, San Diego, CA, USA) according to the Illumina protocol. FASTQ files were generated using the bcl2fastq software (Bcl2Fastq; Illumina). FASTQ data was deposited in GEO (GSE288552)^23^.

### RNA-seq data analysis

Quality control of the reads was assessed with FastQC (version 0.12.0)^28^. Removal of Illumina adapter sequences and trimming of the reads with a phred + 33 quality score below 20 was done using BBDuk (BBMap version 38.90)^29^. Reads were mapped to the *T. solium* genome (assembly GCA_001870725.1)^13^ using the default settings of RSubread (version 2.17.4)^30^. Gene abundance was calculated with GenomicFeatures (v. 1.52.1)^31^ using predicted gene annotations available in the WormBase ParaSite database (version WBPS18)^13,32^.

Variance-stabilized counts were calculated using a generalized linear model that assumes a negative binomial distribution of count data, implemented by DESeq 2 (v.1.40.2)^33^, and were used for a principal component analysis (PCA) using the 500 genes with the highest variance. Additionally, hierarchical clustering was performed across all sample counts by calculating a Euclidean distance-based matrix.

### Functional annotation of the *T. solium* genome

We retrieved full-length *T. solium* transcript sequences from the WormBase Parasite database (PRJNA170813)^13,32^. After importing the sequences into Blast2GO (OmicsBox suite)^24^, we replaced duplicate IDs and added descriptions. A DIAMOND blastx search was conducted against the NR database with an E-value of 1 × 10^−^^5^ and a taxonomy filter for Platyhelminthes (6157), Trematoda (6178), Cestoda (6199), and Nematoda (6231).

We used InterProScan^34^ version 5.69–101.0 (July 2024) to identify conserved domains, motifs, and signal peptides, applying various databases; see Table 1. High-confidence functional annotations were assigned using an E-value threshold of 1 × 10^−^^6^, an annotation cutoff of 55, and a GO weight of 5. Additionally, we integrated functional annotations from eggNOG 5^35^ to enhance annotation quality with orthologous group information using a seed ortholog E-value filter of 1 × 10^−^^5^ and a seed ortholog bit-score filter of 60.0.

**Table 1:** Summary of the InterProScan results obtained for gene function annotation with the Blast2GO suite.

Database	Total # of hits	Total # of unique hits
CATH-GENE3D (Protein families and domain architectures)	12030	1656
PANTHER (Gene/protein families and functional classification)	7350	3735
PFAM (Protein families and domains)	10189	3623
PHOBIUS (Transmembrane and signal peptides prediction)	22540	7
PRINTS (Protein family ‘fingerprints’ - groups of conserved motifs)	1670	428
PROSITE_PROFILES (Protein families, domains and functional sites - position specific score matrices)	7553	702
TMHMM (Transmembrane helices prediction)	6630	1
FUNFAM (Protein structural domains)	3362	2318
SUPERFAMILY (Protein structural and functional annotation)	9639	865
SMART (Signaling and modular protein domains)	4578	728
CDD (Conserved domains in protein sequences)	3736	1611
PROSITE_PATTERNS (Protein families, domains and functional sites - short sequence motifs)	3003	420
COILS (Coiled-coil domain prediction)	3187	1
PIRSR (HMMs and rules to match sites, such as catalytic sites, and binding sites)	14279	703
SIGNALP (Signal peptide prediction)	1292	5
NCBIFAM (Curated protein families)	504	326
PIRSF (Protein families based on evolutionary relationships)	410	301
HAMAP (Conserved protein families or subfamilies)	208	182
SFLD (Sequence-structure-function relationships)	60	22
ANTIFAM (Spurious or misannotated protein families)	1	1

The total number of hits and unique hits for each database across 12,356 *T. solium* coding sequences retrieved from WormBase ParaSite (PRJNA170813) are shown.

Finally, we exported the annotated data into R^36^ (version 4.4.2), creating a custom annotation package using the makeOrgPackage() function from AnnotationForge^25^ for downstream analyses. This process provides a solid framework for exploring the functional roles of *T. solium* genes across different conditions.

## Data Records

This dataset consists of bulk RNA-seq data from 21 samples, representing seven experimental groups, each with three biological replicates. Each sample comprises four pooled *Taenia solium* cysts. The groups include non-cultured parasites (NC); non-evaginated parasites cultured for 6 h without TA (PRE TA-) or with TA (PRE TA+); early-evaginated parasites cultured between 24 and 48 h without TA (EV TA-) or with TA (EV TA+); and fully evaginated parasites cultured for 120 h without TA (POST TA-) or with TA (POST TA+). The raw sequencing data and gene counts matrix are available in the Gene Expression Omnibus (GEO) under accession number GSE288552^23^, while the *T. solium* genome can be accessed via WormBase ParaSite^13,32^.

Scripts and data used for validating and analyzing the transcriptome of *T. solium* activated cysts, which will allow the users interested in the dataset to explore and mine data to generate hypotheses, are available at Zenodo^37^ (10.5281/zenodo.14814788). The pipeline includes quality control, trimming, read mapping, and count matrix generation for validating read quality and mapping to the *T. solium* reference genome. It also features clustering analysis for experimental group validation, and functional annotation. Data analysis scripts cover tasks such as PCA, hierarchical clustering, and functional annotation with the corresponding files for a custom annotation package. In addition, this repository contains a summary table of mapped reads (table_reads_summary.xlsx), raw count matrices (counts.RData), the DESeq 2 object (dds.RData), and functional annotations (Tsolium_annotation_full-export.xlsx).

## Technical Validation

Working permanently in aseptic conditions (clean environments and disinfected or sterile materials) in Tumbes and Lima reduced contamination risk. Veterinarians used protective personal equipment to extract cysts from pig muscle tissue, and surfaces were disinfected with 70° alcohol. Plastic materials were sterile or disinfected, and metal or glass materials were autoclaved. Cysts were extracted with new, sterile scalpel blades, and placed in sterile glass Petri dishes with cold transport solution during the 2–3 h collection procedure. This solution was prepared by autoclaving 1X PBS, adding antibiotics after cooling down, and filtering through 0.22 μm filters. All cysts were inspected to ensure that they were free from any host’s remnants. The transport solution was replaced between all washes done in both laboratories. After culture, special care was taken to keep aseptic conditions. All cultured cysts (elongated vesicles, approximately 1 cm long) looked healthy, with a smooth surface and clear content. The removal of the vesicle and its contents, as well as washing the resulting “juvenile” worm, meant that RNA was extracted only from the actual tissue involved in development and growth, avoiding background from senescent or decaying tissue, as well as contamination from any improbable pig material that could have been absorbed by the cyst before being excised from its host and had remained viable through all the processes.

When measured by NanoDrop, the A260/A280 ratios for RNA were between 1.8 and 2.0. Additionally, the 18S and 28S ribosomal RNA bands were clearly visible in total RNA run on denaturing 1.5% agarose gel. All 21 libraries run on a 2% agarose gel showed a minimum size of ~250 bp in length. We estimated that our 21 libraries could be sequenced with one cartridge using the NextSeq 550 system with the High Output kit v2.5 from Illumina (Illumina, U.S.A.). Based on the human genome (3.1 billion base pairs), the commercial recommendation is 50 million reads for a transcriptome (16 transcriptomes with one cartridge). Considering that the *T. solium* genome (around 122 million base pairs) is 25 times smaller than the human genome, we estimate that 30 million reads are enough to cover each of the transcriptomes we wish to compare. Therefore, 400 million reads allowed sequencing of the seven groups’ biological triplicates (i.e., 19 million reads per transcriptome). These triplicates are also based on pools of individuals, giving more power to the output.

**Fig. 2:**
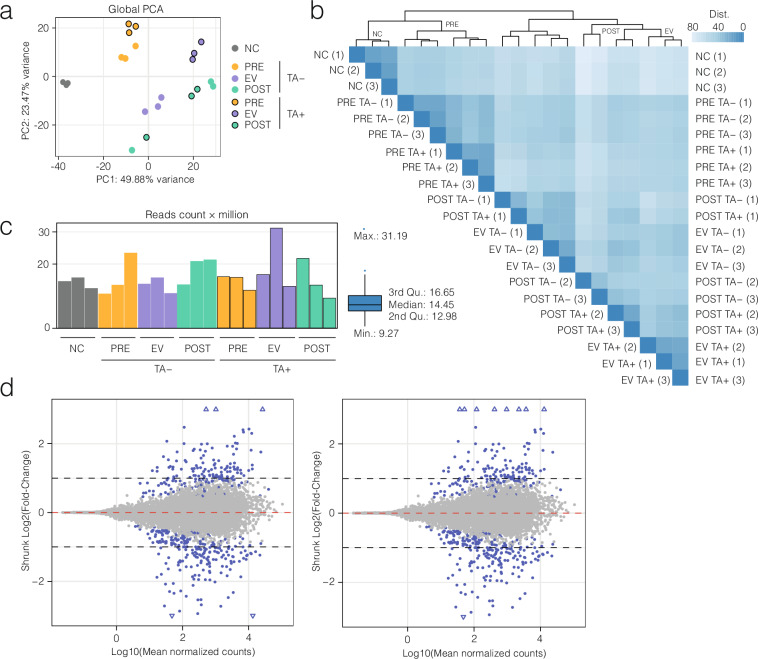
Quality control and validation analysis of transcriptomic data across cyst developmental phases and TA treatment. (**a**) Principal Component Analysis (PCA) plot showing global transcriptomic variation among samples. Samples cluster primarily by parasite group and phase (represented by colors) and secondarily by TA treatment (denoted by outlined circles). (**b**) Hierarchical clustering heatmap of sample-to-sample distances based on variance-stabilized transformed counts. Clustering reflects developmental phases and TA conditions, supporting the consistency of sample grouping. (**c**) Sequencing depth (mapped reads) per replicate, with or without TA treatment (bar plot) and overall distribution (boxplot). Read counts range from approximately 9 to 31 million per sample, with a median of ~14.5 million. (**d**) MA plots displaying the shrunk log_2_ fold-change relative to the log_10_ of mean normalized counts, comparing EV vs. PRE in the absence (left) and presence (right) of TA.

Data replicability was assessed by performing a principal component analysis (PCA) on the counts per sample for the 500 most variable genes (Fig. 2a). The first two principal components (PCs) accounted for 73.35% of the total variance. PCA revealed clustering of biological replicates according to their experimental condition, except for POST parasites in both TA- and TA+, likely reflecting higher variability due to prolonged *in vitro* conditions. Notably, EV parasites, whether stimulated or not with TA, formed two distinct clusters. PRE parasites clustered together and apart from NC, EV, and POST parasites. Clustering indicates that both group/phase and TA influence gene expression profiles.

Hierarchical clustering of samples (Fig. 2b) further highlighted a single cluster encompassing NC and PRE parasites. PRE parasites formed two subclusters based on treatment, while NC and PRE parasites were clearly distinct in gene expression from EV and POST parasites. Additionally, the impact of TA on gene expression in EV cysts was evident, as they formed two well-differentiated subclusters based on treatment. Within the EV-POST cluster, two replicates of POST TA- and two replicates of POST TA + grouped with the EV TA + parasites, while one POST TA- and one POST TA + replicate clustered with the EV TA- group, pointing at transcriptional heterogeneity among the POST parasites.

After quality control with FastQC, only 8.60 ± 5.61% of the reads had to be trimmed due to quality adapter sequence contamination and low read quality (phred + 33 < 20). An average of 15.89 million mapped reads were counted for each replicate. In addition to that, almost all the detected reads (98.47 ± 0.19%) were uniquely mapped to the reported *T. solium* genome (Fig. 2c).

As an example of pairwise comparison, we generated MA plots of the shrunk fold changes between EV and PRE parasites (±TA). These indicate that most transcripts have mean normalized counts above 10, preventing quantification bias due to high variance. Thus, DEGs will be detected by applying a log_2_(fold change) threshold greater than 1 or less than −1 (Fig. 2d).

## Data Availability

Scripts and files used for processing, analysis, and visualization of this transcriptome have been deposited in Zenodo (10.5281/zenodo.14814788)^37^.
